# Insights into Actin Isoform-Specific Interactions with Myosin via Computational Analysis

**DOI:** 10.3390/molecules29132992

**Published:** 2024-06-23

**Authors:** Chan Jong Yu, Yoon Ho Park, Mi Young An, Bumhan Ryu, Hyun Suk Jung

**Affiliations:** 1 Department of Biochemistry, College of Natural Sciences, Kangwon National University, Chuncheon 24341, Gangwon, Republic of Korea; cjyu@kangwon.ac.kr (C.J.Y.); yhpark99@kangwon.ac.kr (Y.H.P.); myan@kangwon.ac.kr (M.Y.A.); 2 Research Solution Center, Institute for Basic Science, Daejeon 34126, Republic of Korea

**Keywords:** actin isoforms, sequence alignment, protein complex prediction, loop 2, actomyosin complex

## Abstract

Actin, which plays a crucial role in cellular structure and function, interacts with various binding proteins, notably myosin. In mammals, actin is composed of six isoforms that exhibit high levels of sequence conservation and structural similarity overall. As a result, the selection of actin isoforms was considered unimportant in structural studies of their binding with myosin. However, recent high-resolution structural research discovered subtle structural differences in the N-terminus of actin isoforms, suggesting the possibility that each actin isoform may engage in specific interactions with myosin isoforms. In this study, we aimed to explore this possibility, particularly by understanding the influence of different actin isoforms on the interaction with myosin 7A. First, we compared the reported actomyosin structures utilizing the same type of actin isoforms as the high-resolution filamentous skeletal α-actin (3.5 Å) structure elucidated using cryo-EM. Through this comparison, we confirmed that the diversity of myosin isoforms leads to differences in interaction with the actin N-terminus, and that loop 2 of the myosin actin-binding sites directly interacts with the actin N-terminus. Subsequently, with the aid of multiple sequence alignment, we observed significant variations in the length of loop 2 across different myosin isoforms. We predicted that these length differences in loop 2 would likely result in structural variations that would affect the interaction with the actin N-terminus. For myosin 7A, loop 2 was found to be very short, and protein complex predictions using skeletal α-actin confirmed an interaction between loop 2 and the actin N-terminus. The prediction indicated that the positively charged residues present in loop 2 electrostatically interact with the acidic patch residues D24 and D25 of actin subdomain 1, whereas interaction with the actin N-terminus beyond this was not observed. Additionally, analyses of the actomyosin-7A prediction models generated using various actin isoforms consistently yielded the same results regardless of the type of actin isoform employed. The results of this study suggest that the subtle structural differences in the N-terminus of actin isoforms are unlikely to influence the binding structure with short loop 2 myosin 7A. Our findings are expected to provide a deeper understanding for future high-resolution structural binding studies of actin and myosin.

## 1. Introduction

Actin, a cytoskeletal protein expressed ubiquitously in many eukaryotic cells, consists of 375–377 amino acids [1]. Monomeric actin has a globular structure with four subdomains. Subdomains 1 and 3 are structurally related, whereas subdomains 2 and 4 consist of large insertions into subdomains 1 and 3. Subdomains 1 and 2 form the more exposed outer domain, and subdomains 3 and 4 constitute the inner domain. Actin contains two opposite clefts, with one located between subdomains 2 and 4 among the nucleotide-binding sites, and the other situated between subdomains 1 and 3, where they mediate most interactions with actin-binding proteins [1,2,3]. Monomeric or globular actin forms filamentous actin through nucleation, elongation, and steady-state processes [4,5,6]. The isoforms of filamentous actin are involved in maintaining the cell shape, cell division, exocytosis, endocytosis, secretion, signal transduction, and the regulation of enzyme activities [7,8,9]. The functional diversity of actin arises from its expression timing, localization within a cell, and interactions with numerous actin-binding proteins [10,11]. Actin expressed in various tissues and cells is encoded by individual genes, with humans expressing six actin genes in a tissue-specifically regulated manner [12,13]. Among these, four are muscle actins (skeletal α-actin, cardiac α-actin, smooth α-actin, smooth γ-actin), and two are non-muscle actins (cytoplasmic γ-actin, cytoplasmic β-actin). These actin isoforms share a high sequence identity [14,15,16]. Despite the high overall sequence similarity across the actin isoforms, the sequences vary at the N-terminus. These variations are predominantly composed of negatively charged residues, but these vary between isoforms, with cytoplasmic γ-actin and cytoplasmic β-actin exhibiting the greatest differences in these sequences [17,18]. Actin isoforms perform specific and overlapping roles within cells by engaging in diverse biochemical functions, cellular localization, and interactions with actin-binding proteins. Differences between actin isoforms facilitate the formation of diverse actin networks within cells to enable various functions [19,20,21].

Research on the structure of actin, initiated around 30 years ago, has continued actively to this day with researchers employing X-ray crystallography and cryo-electron microscopy (cryo-EM) techniques to investigate the various actin isoforms. Among the six isoforms, while the structures of smooth α-actin and γ-actin remain unknown, those of skeletal α-actin, cardiac α-actin, cytoplasmic γ-actin, and cytoplasmic β-actin have been elucidated [18,22,23,24,25]. Of these isoforms, investigations into the structure and function of skeletal α-actin have been the most actively pursued, and this isoform is employed in the majority of studies involving actin-binding protein complexes [26,27,28,29,30,31]. Among the actin isoforms, the overall structure generally has a conserved architecture, with minimal variation observed in the lateral and longitudinal interfaces or the pitch of the actin helix [18,32]. However, recent cryo-EM structural studies have revealed subtle structural differences among the N-termini of the actin isoforms, suggesting that these differences may contribute to the formation of isoform-specific interactions with actin-binding proteins [18,33,34].

Myosin is one of the prominent actin-binding proteins, with numerous roles such as muscle contraction, intracellular transport, and cell movement by binding to filamentous actin and facilitating movement. Myosin is a motor protein composed of numerous isoforms. Extensive functional studies over a long period have revealed a wealth of information about the roles of these isoforms [35,36,37]. For example, unconventional myosin 7A plays a crucial role in the growth and maintenance of hair bundles in stereocilia. Mutations in this gene lead to Usher syndrome type 1B, a genetic disorder where affected infants experience profound hearing loss or complete deafness. Currently, there is no cure for Usher syndrome, and management relies on hearing aids, cochlear implants, visual aids, and similar devices. Functional studies using shaker-1 mice with mutated myosin 7A genes have demonstrated the importance of this gene in the arrangement of hair bundles in stereocilia and the survival of retinal cells [38,39,40,41,42].

Myosin, composed of motor, neck, and tail domains, undergoes movement via a mechanism where, upon binding with ATP, hydrolysis occurs. This leads to the conversion to the ADP-Pi state by forming a cross-bridge with the filamentous actin, and the power stroke step occurs upon the release of Pi [43,44,45,46,47]. The binding sites of myosin that interact with filamentous actin are classified into five main regions within the motor domain: loop 4, the cardiomyopathy loop, the helix–loop–helix motif, loop 3, and loop 2 [48,49,50,51]. Among these, loop 2 is known to induce initiation binding by forming interactions with subdomains 1 and 3 [52,53,54,55,56,57]. In a 2016 paper on the human cytoplasmic actomyosin complex, it was proposed that in the ADP-Pi state, loop 2 engages in weak binding with subdomain 1 and subdomain 3 of actin, whereupon it is attracted towards the actomyosin interface via electrostatic interactions with the actin N-terminus, thereby stabilizing a large-scale attraction mechanism [48]. However, loop 2 reportedly exhibits the greatest variability in its sequence and structure among the isoforms within the motor domain of myosin [55]. For instance, although myosin isoforms such as non-muscle myosin 2C have a long loop 2, other isoforms such as myosin 1B have a very short loop 2 [29,48]. These differences in the length of loop 2 may influence interactions with the actin N-terminus.

Current cryo-EM technology is capable of resolving the atomic-level structure of actomyosin complexes to provide a structural understanding of the complex that forms between actin and myosin [18,24,25,28,29,30,31]. However, due to the extensive time and effort required for the entire process of structural determination, this study investigated the potential structural impact of the differences in the actin isoforms on myosin 7A by conducting sequence-based computational analysis. Specifically, the focus was on obtaining information about the interaction between loop 2 of myosin 7A and the actin N-terminus using sequence alignment and protein complex prediction. Using these methods, loop 2 of myosin 7A was predicted to be very short, and therefore, interaction with the actin N-terminus would not be possible. The results of this study suggest that subtle structural differences in the N-terminus of actin isoforms may have minimal structural impacts on the interaction with myosin 7A. 

## 2. Results

### 2.1. Variations in Sequence and Structure Depending on Actin Isoforms

**Figure 1:**
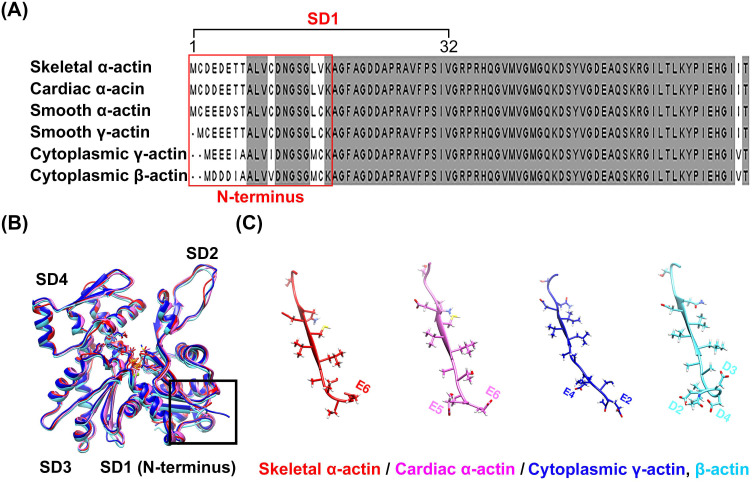
Comparison of the amino acid sequence and model of the N-terminus of actin isoforms. Sequence alignment (**A**) of the six isoforms of actin (Supplementary Figure S1), where the N-terminus acidic residue exhibits the greatest sequence diversity. Conserved residues are highlighted in gray. Superimposition of the structures of the four elucidated actin isoforms (**B**) revealed structural differences among the isoforms at the N-terminus (**C**). The models (**B**,**C**) were extracted from the deposited structure of bare actin isoforms [18], i.e., skeletal α-actin (PDB: 8DMX, red), cardiac α-actin (PDB: 8DMY, magenta), cytoplasmic γ-actin (PDB: 8DNF, blue), and cytoplasmic β-actin (PDB: 8DMH, cyan).

**Figure 2:**
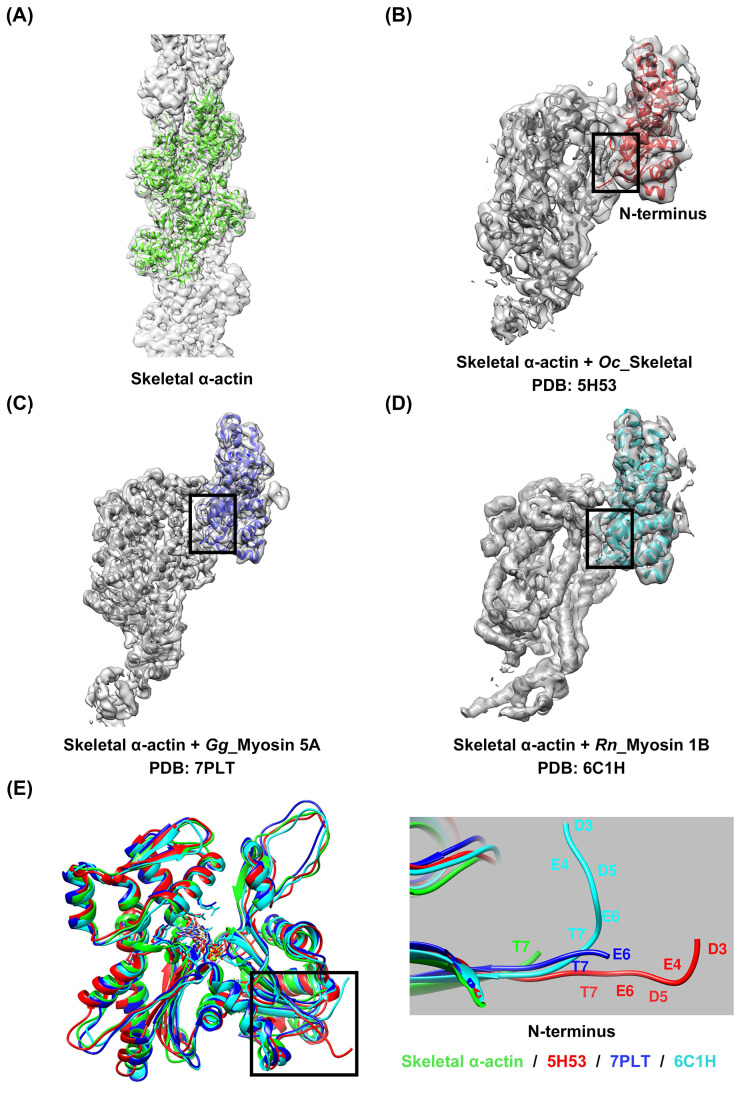
Comparison of actin structures among models derived using skeletal α-actin. (**A**) Helical reconstruction of skeletal α-actin using cryo-EM (Supplementary Figure S2, Supplementary Table S1). The models (**B**–**D**) were extracted from the deposited actomyosin complex structures, i.e., actomyosin-2 skeletal (PDB: 5H53) [26], actomyosin-5A (PDB: 7PLT) [30], and actomyosin-1B (PDB: 6C1H) [29]. (**E**) Superimposition of models (**A**–**D**) for structural comparison revealed differences in orientation at the N-terminus, even for the same actin isoform. Skeletal α-actin (cryo-EM): green; skeletal actomyosin-2: red; actomyosin-5A: blue; actomyosin-1B: cyan.

Despite a high degree of sequence conservation among the actin isoforms, differences in the sequence were observed in the N-terminus, particularly in subdomain 1, where a high distribution of acidic residues prevails (Figure 1A and Supplementary Figure S1). A comparison of the structures of the four isoforms of actin, which were identified by superimposing them, also revealed differences in length and directionality at the N-terminus, which contains many acidic residues (Figure 1B,C). These differences suggest the possibility of isoform-specific interactions with myosin, which could lead to specific interactions with the actin isoforms [18]. However, interactions of this nature have not been reported in any actomyosin complex model studied to date. Instead, isoform-specific interactions with actin have been reported depending on the myosin isoforms, suggesting that myosin isoforms also exert structural influences on the interaction between actin and myosin. We resolved the high-resolution structure of skeletal α-actin (3.5 Å) in the filamentous state using cryo-EM (Figure 2A and Supplementary Figure S2, Supplementary Table S1), and subsequently compared the structures by superimposing the actin from actomyosin complex models constructed using skeletal α-actin (Figure 2B–D). Despite using the same actin isoform, we observed differences in the orientation of the actin N-terminus (Figure 2E). This confirms that myosin isoforms can influence the structural binding between actin and myosin. 

### 2.2. Diversity in Sequence and Length of Myosin Loop 2 Interacting with Actin N-Terminus

**Figure 3:**
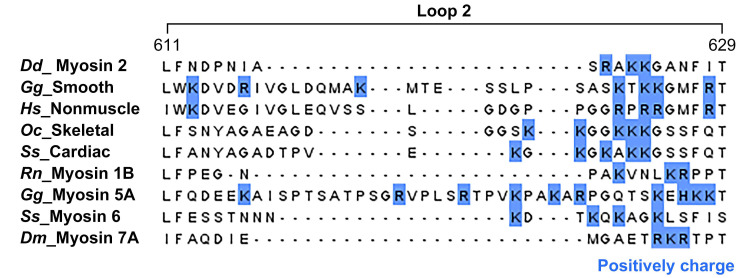
Multiple sequence alignment of the actin-binding sites on loop 2 from myosin 7A and other myosin isoforms. Sequence alignment with the myosin isoforms enabled loop 2 of myosin 7A to be predicted as the binding site for the acidic patch residues, including the N-terminus of actin subdomain 1 among its five major actin-binding sites. Additionally, the sequence length of myosin 7A loop 2 was found to be similar to that of myosin 1B (Supplementary Figure S3). Positively charged residues are indicated in blue. Species codes: *Dd, Dictyostelium discoideum*; *Gg, Gallus gallus*; *Hs, Homo sapiens*; *Oc, Oryctolagus cuniculus*; *Ss, Sus scrofa*; *Rn, Rattus norvegicus*; *Dm, Drosophila melanogaster*.

Loop 2 is known as one of the myosin actin-binding sites that induces the initial interaction between actin and myosin through electrostatic interaction with the acidic patch residues, including the N-terminus of the actin subdomain 1 [52,53,54,55,56,57]. However, loop 2 is the most variable part of the myosin head, with its length varying among the different myosin isoforms [55]. This variability can have significant structural implications for binding with actin. Therefore, we performed multiple sequence alignment using sequences of various myosin isoforms for which actomyosin structural studies have been completed, along with the myosin 7A sequence, to predict the form of loop 2 of myosin 7A (Figure 3 and Supplementary Figure S3). This loop is considerably shorter compared to the typical long loop 2 found in non-muscle myosin 2C [48], in that it resembles the sequence length of myosin 1B [29], which possesses a short loop 2. Therefore, myosin 7A was expected to have a short loop 2, and was predicted to undergo interactions with actin similar to those of myosin 1B loop 2.

### 2.3. Prediction of Specific Interactions between Actin and Loop 2 of the Actomyosin-7A Complex

**Figure 4:**
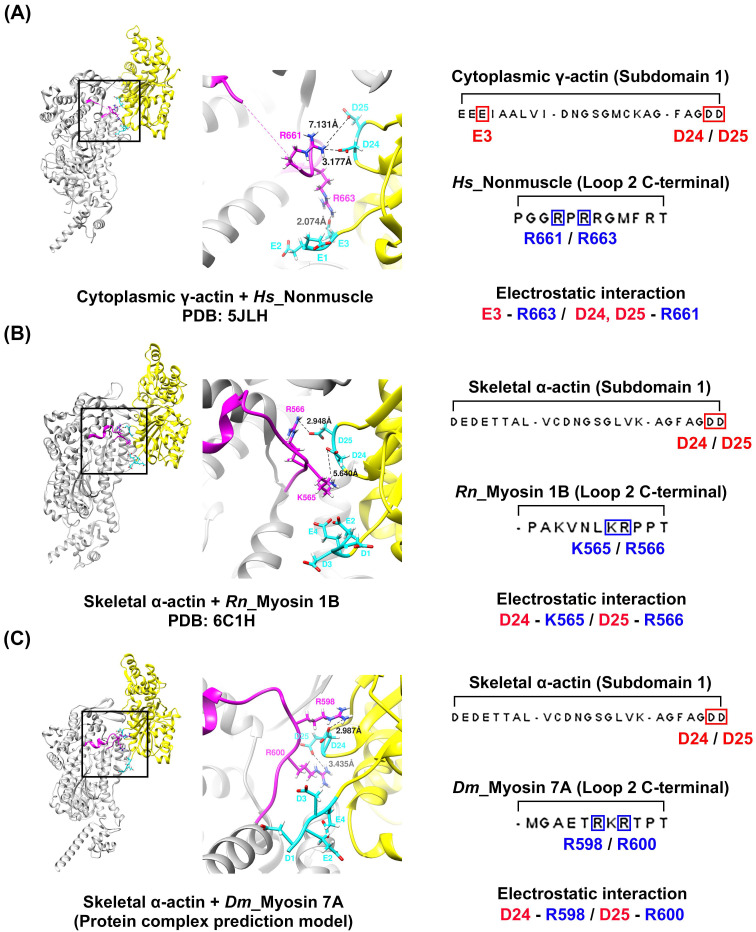
Predicted actin–loop 2 interface of the actomyosin-7A complex. Residues R663 and R661 located at the C-terminus of the long loop 2 in non-muscle myosin 2C establish electrostatic interactions with actin N-terminus E3 and actin subdomain 1 acidic patch residues D24/D25, respectively (**A**). On the other hand, myosin 1B (**B**) and myosin 7A (**C**), which have a short loop 2, do not additionally interact with the actin N-terminus apart from the electrostatic interaction with the acidic patch residues D24 and D25 of actin subdomain 1 at the C-terminus of loop 2. The models (**A**,**B**) were extracted from the deposited actomyosin complex structures, i.e., actomyosin-2 non-muscle (PDB: 5JLH) [48], actomyosin-1B (PDB: 6C1H) [29]. (**C**) The model utilized in this study was constructed using the same procedure as that of the actomyosin-7A structural prediction method that was recently reported [58] (AlphaFold2_multimer 2.3.1 version software). Positively and negatively charged residues are displayed in red and blue, respectively. Actin: yellow; actin N-terminus acidic residue: cyan; loop 2: magenta. Species codes: *Hs, Homo sapiens*; *Rn, Rattus norvegicus*; *Dm, Drosophila melanogaster*.

**Figure 5:**
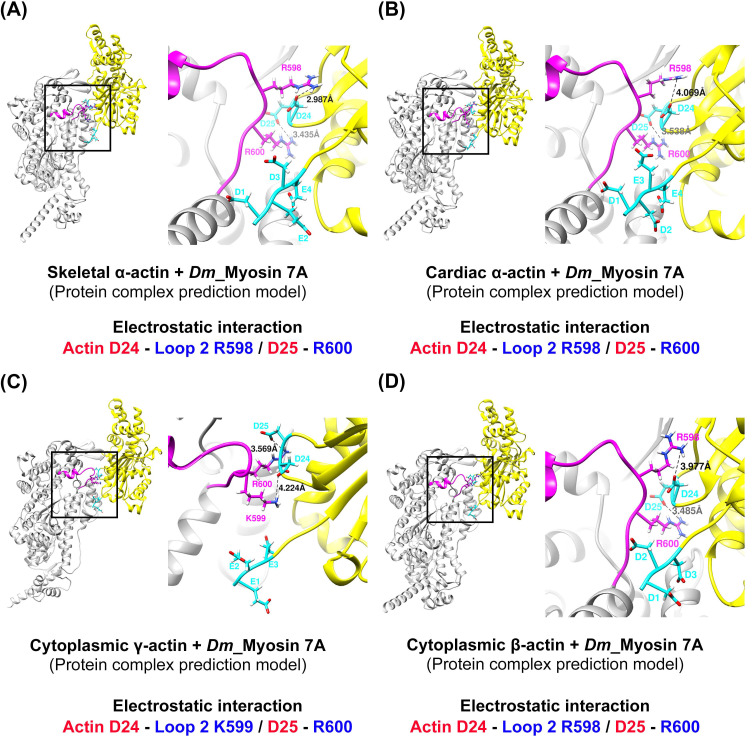
Prediction of the actin–loop 2 interface in actomyosin-7A complexes with different types of actin isoforms. (**A**–**D**) The comparison of four different actomyosin-7A prediction models, based on varying actin isoforms, revealed that the three positively charged residues (R598–R600) at the loop 2 C-terminus electrostatically interact with actin subdomain 1 acidic patch residues D24 and D25. No additional interaction with the actin N-terminus was observed. (**A**–**D**) The model utilized in this study was constructed using the same procedure as for the structural prediction of actomyosin-7A [58] (AlphaFold2_multimer 2.3.1 version software). Positively and negatively charged residues are displayed in red and blue, respectively. Actin: yellow; actin N-terminus acidic residue: cyan; loop 2: magenta. Species codes: *Dm, Drosophila melanogaster*.

To explore the potential interactions between loop 2 of myosin 7A, predicted to be short, and the actin N-terminus, we constructed a model of the actomyosin-7A complex using the same protein–protein interaction approach as was recently employed for predicting actomyosin structures [58] (Supplementary Figure S4). We compared and analyzed the model based on the predicted actomyosin-7A, the representative non-muscle myosin 2C with a long loop 2 [48], and the myosin 1B actomyosin model with a short loop 2 [29] (Figure 4). In the case of non-muscle myosin 2C with a long loop 2, residues with a positively charged C-terminus in loop 2 not only engage in electrostatic interactions with the actin subdomain 1 acidic patch residues D24 and D25 but also with additional actin N-terminus E3 (Figure 4A). In contrast, myosin 1B with a short loop 2 does not participate in additional interactions with the actin N-terminus beyond those with actin subdomain 1 acidic patch residues D24 and D25 by residues with a positively charged C-terminus in loop 2 (Figure 4B). The positively charged residue that is present in the C-terminus of loop 2 in myosin 7A also does not take part in additional interactions with the actin N-terminus, apart from the electrostatic interaction with actin subdomain 1 acidic patch residues D24 and D25 (Figure 4C). Furthermore, we generated additional models of the actomyosin-7A complex using various actin isoforms and analyzed the interaction between loop 2 and the N-terminus of each actin isoform. During this process, we observed no additional interactions beyond the electrostatic interaction with actin subdomain 1 acidic patch residues D24 and D25. Considering the potential structural flexibility of loop 2, we conducted molecular dynamics simulations and confirmed that the results are consistent (Figure 5 and Supplementary Figure S5). Based on these results, the specific interactions of myosin 7A are not predicted to be governed by the actin isoforms.

## 3. Discussion

Actin isoforms exhibit high levels of sequence conservation and structural similarity, but recent high-resolution cryo-EM studies reported structural differences in the N-terminus depending on the actin isoforms [18]. Despite extensive research on the binding structure of actin and myosin over many years, our understanding of the structural influence exerted by the type of actin isoform on the interaction with myosin was insufficient. This motivated our study, which focused on expanding this knowledge by utilizing sequence alignment and protein complex prediction techniques, particularly to explore the impact of subtle structural differences among the actin isoforms on the binding structure with myosin 7A.

Myosin participates in weak binding with filamentous actin during the initial stages of the ATPase cycle, where the acidic residues of the actin N-terminus and subdomain 1 interact with loop 2, one of the actin-binding sites of myosin [52,53,54,55,56,57]. However, loop 2 is the most variable characteristic in the myosin motor domain, with its length varying by more than 100 amino acids [59,60,61]. These differences can have a significant influence on the way in which it binds with actin. For example, in the case of myosin 1B with a short loop 2, the positively charged C-terminal of loop 2 interacts with the acidic patch in actin subdomain 1, but does not interact with the actin N-terminus [18]. However, in the case of non-muscle myosin 2C with a long loop 2, additional interactions with the actin N-terminus are possible [48]. The results of sequence alignment suggest that the sequence length of myosin 7A loop 2 is short and is expected to form initial binding with actin similar to myosin 1B, which has a representative short loop 2 (Figure 3).

All myosin isoforms possess positively charged residues at the C-terminal of loop 2, which electrostatically interact with the acidic patch residues D24 and D25 of actin subdomain 1 [28,29,30,31,48,49]. This interaction is observed for all actomyosin structures, and studies concerned with the mutation of the positively charged C-terminal of loop 2 have found this interaction to be essential to initiate the binding between actin and myosin [54,55,62,63]. However, the electrostatic interaction between the N-terminus and loop 2 of actin is observed in myosin isoforms with a long loop 2, where, to stabilize the structure, the acidic residues of the actin N-terminus induce electrostatic interaction with the positive charges of loop 2 when Pi is released [53]. Analysis of the predicted actomyosin-7A structure revealed that some of the three positive charges at the C-terminal of loop 2 engaged in electrostatic interactions with actin D24 and D25, and that additional interactions with the actin N-terminus did not take place (Figure 4 and Figure 5). Myosin with a short loop 2, which includes myosin 7A, is predicted to have limited ability to induce structural stabilization by electrostatically interacting with the actin N-terminus due to geometric constraints.

In conclusion, the combination of structural and functional studies on the relationship between actin and myosin in our research led us to propose that the influence of structural differences in the N-terminus of actin isoforms on the structural interaction with myosin is highly dependent on the length of myosin loop 2. In particular, the impact on myosin isoforms with a short loop 2 is expected to be particularly limited. Using in silico experiments, we have provided an in-depth understanding of the complex interactions between actin isoforms and myosin, but the limitations of computational analysis primarily lie in the simplification or idealization of complex biological systems. For example, because computational analysis may not fully reflect various factors in the actual biological environment, it is necessary to verify whether it aligns with our predicted results through additional verification using experimental data such as Cryo-EM. This will contribute to a more robust understanding of the interactions between actin and myosin within biological systems.

## 4. Materials and Methods

### 4.1. Multiple Sequence Alignment and Analysis

Whole-protein sequences of actin and myosin isoforms were downloaded from the UniProt database “https://www.uniprot.org/ (accessed on 12 March 2024)” : skeletal α-actin (*Oryctolagus cuniculus*, UniProt-P68135), cardiac α-actin (*Bos taurus*, UniProt-Q3ZC07), smooth α-actin (*Homo sapiens*, UniProt-P62736), smooth γ-actin (*Homo sapiens*, UniProt-P63267), cytoplasmic γ-actin (*Homo sapiens*, UniProt-P63261), cytoplasmic β-actin (*Homo sapiens*, UniProt-P60709), myosin 2 (*Dictyostelium discoideu*, UniProt-P08799), myosin 2 smooth (*Gallus gallus*, UniProt-P10587), myosin 2 non-muscle (*Homo sapiens*, UniProt-Q7Z406), myosin 2 skeletal (*Oryctolagus cuniculus*, UniProt-Q9GJP9), myosin 2 cardiac (*Sus scrofa*, UniProt-P79293), myosin 1B (*Rattus norvegicus*, UniProt-Q05096), myosin 5A (*Gallus gallus*, UniProt-Q02440), myosin 6 (*Sus scrofa*, UniProt-Q29122), and myosin 7A (*Drosophila melanogaster*, UniProt-Q9V3Z6). Alignments were generated using T-Coffee software “https://tcoffee.crg.eu/apps/tcofee/do:expresso (accessed on 13 March 2024)” [64] in the ClustalW output format, and then colored using the Jalview program “2.11.3.2, The Barton Group, University of Dundee, Scotland, UK“ [65].

### 4.2. Cryo-EM Sample Preparation and Image Processing

The skeletal α-actin from rabbit skeletal muscle was a generous gift from Ikebe’s laboratory and was stored at −80 °C before use. For cryo-EM studies, skeletal α-actin was polymerized by the addition of buffer containing 50 mM Na-acetate, 2 mM MgCl_2_, 1 mM EGTA, and 10 mM Mops at pH 7.5 before plunge freezing. This buffer was supplemented with an equimolar ratio of phalloidin to actin to stabilize the filaments. Frozen–hydrated specimens were prepared on a glow-discharged (15 mA current, 60 s) holey carbon grid (Quantifoil R 2/2 Cu 200 mesh) using Vitrobot Mark IV (ThermoFisher Scientific Inc. Waltham, MA, USA) at 4 °C and 100% humidity. Cryo-EM images were acquired at the Korea Basic Science Institute (KBSI, Ochang, Republic of Korea), using a Titan Krios G2 instrument (ThermoFisher Scientific Inc. Waltham, MA, USA) with a Falcon 3EC direct electron detector (DED). Automated data acquisition was performed in electron counting mode using EPU 2.6.1 version software (ThermoFisher Scientific, USA). Additional details are provided in Supplementary Table S1. A total of 1882 micrographs were recorded with 40 subframes at a dose rate of ~1.0 e-/Å 2 per frame over a defocus range of −1.4~−2.2 µm with a pixel size of 1.09 Å. Images were imported into Relion 3.1 software [66], where motion correction and dose weighting were accomplished using an internal Relion 3.1 implementation. The contrast transfer function (CTF) was used with Gctf [67]. Particles were auto-picked with Gautomatch “http://www.mrc-lmb.cam.ac.uk/kzhang/Gautomatch/ (accessed on 27 March 2024)”, and 203,610 particles were extracted with a box size of 370 Å and an overlap of 27.89 Å. After 2D classification, 181,143 segments were used for 3D classification. The best 3D class from the dataset was selected, and a low-pass filter was applied to 40 Å to serve as the initial reference for subsequent 3D auto-refinement with helical symmetry. After 3D auto-refinement and postprocessing, maps with 4.8 Å resolution based on the Fourier shell correlation (FSC) 0.143 criterion [68] were obtained. The resolution was further improved with CTF refinement [69] and Bayesian polishing [70]. Subsequent 3D auto-refinement and postprocessing resulted in a final map with a resolution of 3.5 Å (Supplementary Figure S2E) and a helical symmetry of −169.6°/28.3 Å. 

### 4.3. Model Building and Refinement 

The filamentous skeletal α-actin models were built in the Coot program [71,72], with PDB entry 6BNO [28] used as the template. Some of the acidic residues in the actin N-terminus could not be modeled due to their weak density. The structures were refined using phenix.real_space_refine in PHENIX 1.20.1-4487 version software [73] and visualized using UCSF Chimera “1.16, Regents of the University of California, San Francisco, USA” [74].

### 4.4. Model Prediction and Visualization

The structures of the actomyosin-7A complex, which includes Drosophila myosin 7A and four actin isoforms, were predicted by processing each protein sequence with AlphaFold2_multimer 2.3.1 version software [75] using the default settings without template assistance. The evaluation metrics for the generated actomyosin-7A complex model are described in Supplementary Figure S4. GROMACS 2023.3 software package with the CHARMM27 force field [76]. The system was solvated in a cubic solvent box. The solvated system was neutralized by adding ions to achieve an ion concentration of 0.15 M. The charges of the negative and positive ions were set to −1 and 1, respectively. The simulations were carried out for 1 ns. All the proteins in the figures were visualized by UCSF Chimera [74].

## Data Availability

The datasets used and/or analyzed during the current study are available from the corresponding author upon reasonable request.

## Supplementary Materials

The following supporting information can be downloaded at: https://www.mdpi.com/article/10.3390/molecules29132992/s1, Figure S1: Multiple sequence alignment of the six isoforms of actin; Table S1: Cryo-EM data collection, reconstruction statistics, and structure characteristics summary.

## Abbreviations

Cryo-EM (cryo-electron microscopy), ATP (adenosine triphosphate), ADP (adenosine diphosphate), Pi (inorganic phosphate), DED (direct electron detector), EPU (Energy Filtering and Electron Imaging), 3D (three-dimensional), CTF (contrast transfer function), FSC (Fourier shell correlation), MD (molecular dynamic).
