# Supplementary material for: Insights into Actin Isoform-Specific Interactions with Myosin via Computational Analysis
Source: Molecules. 2024 Jun 23;29(13):2992. doi: 10.3390/molecules29132992 (PMC11242942; doi:10.3390/molecules29132992)
Supplement: Supplementary file 1 [file molecules-29-02992-s001.zip › molecules-3068749-supplementary.pdf]

Supplementary Material

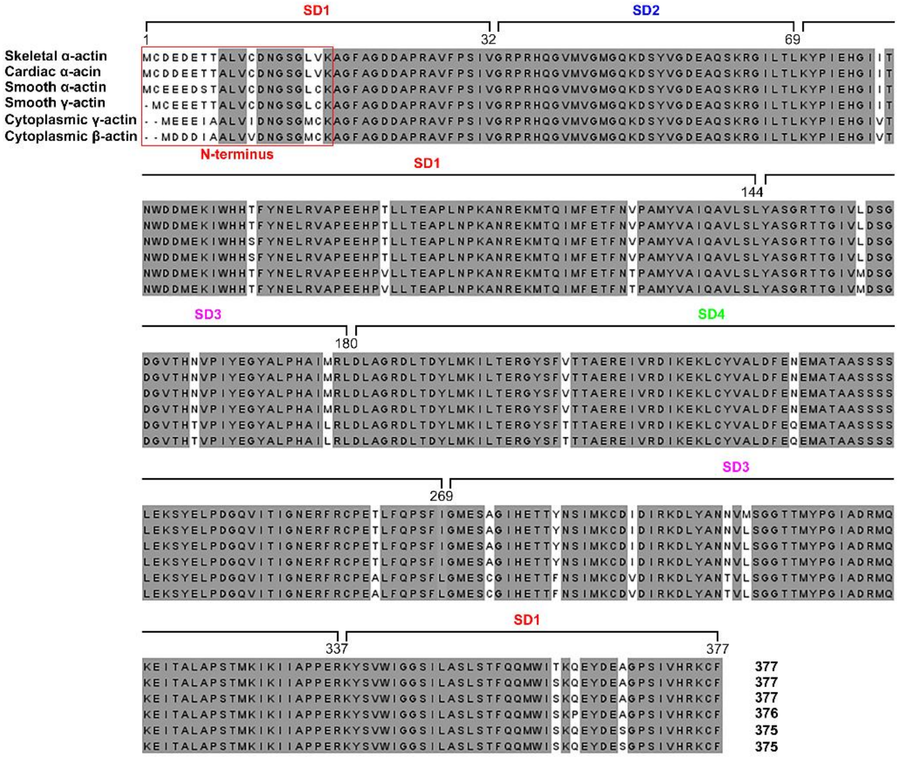

Fig. S1. Multiple sequence alignment of the six isoforms of actin. A high level of amino acid sequence conservation among actin isoforms is observed, but diversity in the sequence is noted in the N-terminus region highlighted in the red box. The shaded areas indicate conserved amino acids. Subdomains (SD) of actin are indicated with different colors. Subdomain 1: Red, subdomain 2: Blue, subdomain 3: Magenta, and subdomain 4: Green.

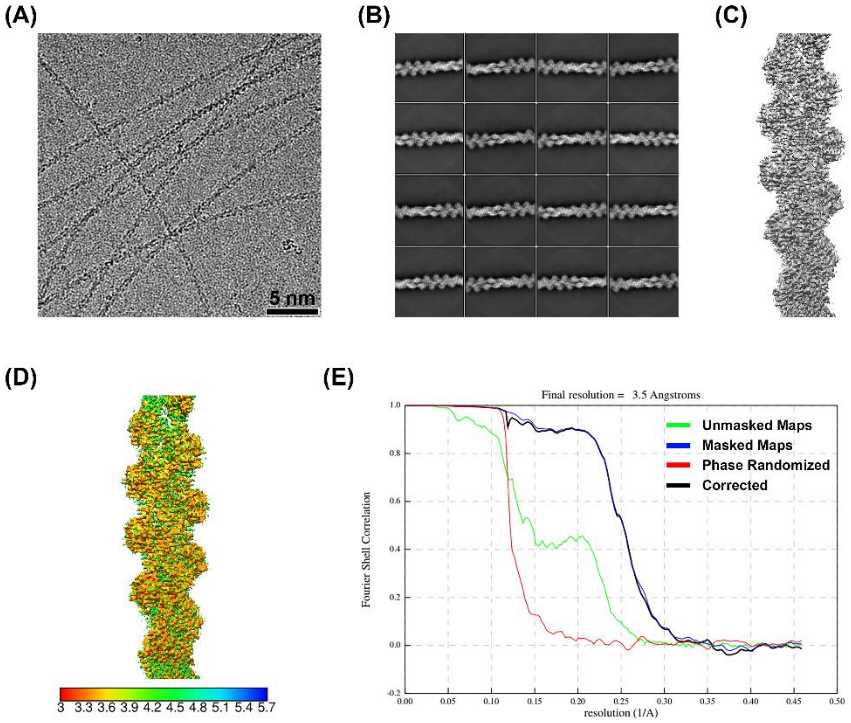

Fig. S2. Details of cryo-EM data processing. Representative images of 1882 micrographs (A), two-dimensional class averages of filamentous actin (B), and the three-dimensional refinement model (C). Estimation of the local resolution of the indicated reconstructions (D). Use of the Fourier shell correlation (FSC) 0.143 criterion to estimate the overall resolution of all 3D reconstructions processed with helical symmetry, as determined by the gold-standard FSC curves (E).

Table S1. Cryo-EM data collection, reconstruction statistics, and structure characteristics summary.

**Map**	**Skeletal alpha-actin**
**Data Collection**	
Microscope	Titan Krios G2
Detector	Falcon III
Acceleration voltage (kV)	300
Nominal magnification	×75,000
Defocus range (μm)	-1.4 ~ -2.2
Total Dose rate (e−/Å2)	40
Pixel size (Å)	1.09
Micrographs	1,882
**Reconstruction**	
Software	RELION 3.1
Box size (pixel)	370
Final no. particle images	181,143
Map Resolution (Å; FSC=0.143)	3.5
Map sharpening B-factor (Å^2)	-81.6396
Symmetry imposed	Helical
**Measured helical symmetry**	
Rise (Å)	28.2658
Helical twist (°)	-169.626
**Structure building and validation**	
Model building	Coot
Refinement program	Phenix
Refinement target	Real-space
**Structures Characteristics**	
Species	Rabbit
Amino acid resolved	7-375

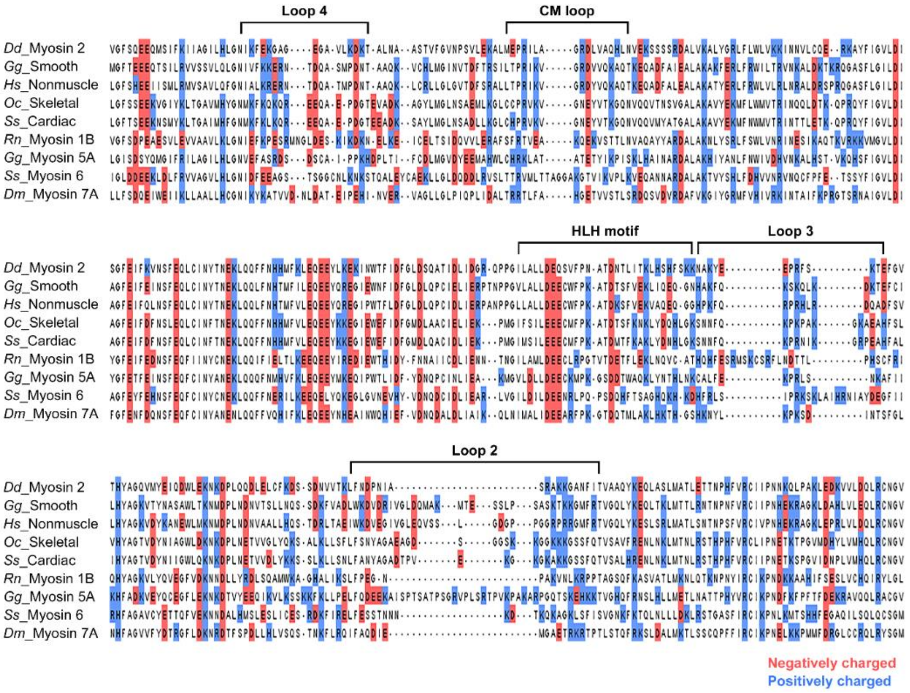

Fig. S3. Amino acid sequence alignment of actin binding sites from myosin 7A and other myosin isoforms. The five major regions known as actin binding sites in myosin: Loop 4, CM loop, HLH motif, Loop 3, and Loop 2. Positively and negatively charged residues are shown in red and blue, respectively. Species codes: *Dd Dictyostelium discoideum; Gg Gallus gallus; Hs Homo sapiens; Oc Oryctolagus cuniculus; Ss Sus scrofa; Rn Rattus norvegicus; Dm Drosophila melanogaster*.

(A)

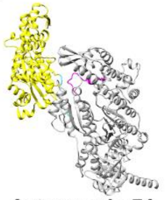

Actomyosin-7A
(Skeletal α-actin)

Model Rank	Mean pLDDT	Max PAE	pTM	ipTM	Uncertainty	Overall Quality
1	90.2	31.44	0.84	0.81	N/A	Very High
2	89.78	31.45	0.83	0.81	N/A	High
3	89.48	31.42	0.84	0.8	N/A	High
4	89.58	31.39	0.82	0.78	N/A	High
5	88.98	31.41	0.81	0.77	N/A	High

Model Confidence
- Very high (pLDDT > 90)
- High (90 > pLDDT > 70)
- Low (70 > pLDDT > 50)
- Very low (pLDDT < 50)

(B)

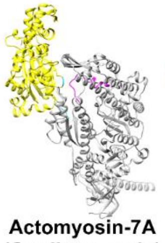

Actomyosin-7A
(Cardiac α-actin)

Model Rank	Mean pLDDT	Max PAE	pTM	ipTM	Uncertainty	Overall Quality
1	90.16	31.44	0.84	0.82	N/A	Very High
2	89.36	31.48	0.82	0.8	N/A	High
3	89.7	31.38	0.83	0.8	N/A	High
4	88.8	31.42	0.82	0.79	N/A	High
5	85.05	31.66	0.62	0.18	N/A	High

Model Confidence
- Very high (pLDDT > 90)
- High (90 > pLDDT > 70)
- Low (70 > pLDDT > 50)
- Very low (pLDDT < 50)

(C)

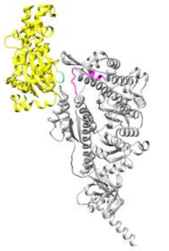

Actomyosin-7A
(Cytoplasmic γ-actin)

Model Rank	Mean pLDDT	Max PAE	pTM	ipTM	Uncertainty	Overall Quality
1	90.1	31.45	0.84	0.83	N/A	Very High
2	89.98	31.48	0.84	0.83	N/A	High
3	89.66	31.44	0.83	0.82	N/A	High
4	89.6	31.44	0.84	0.81	N/A	High
5	85.46	31.66	0.62	0.19	N/A	High

Model Confidence
- Very high (pLDDT > 90)
- High (90 > pLDDT > 70)
- Low (70 > pLDDT > 50)
- Very low (pLDDT < 50)

(D)

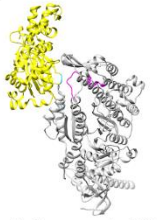

Actomyosin-7A
(Cytoplasmic β-actin)

Model Rank	Mean pLDDT	Max PAE	pTM	ipTM	Uncertainty	Overall Quality
1	90.47	31.48	0.85	0.83	N/A	Very High
2	89	31.39	0.82	0.81	N/A	High
3	89.22	31.47	0.82	0.81	N/A	High
4	89.67	31.44	0.83	0.8	N/A	High
5	90.42	31.41	0.84	0.79	N/A	Very High

Model Confidence
- Very high (pLDDT > 90)
- High (90 > pLDDT > 70)
- Low (70 > pLDDT > 50)
- Very low (pLDDT < 50)

Fig. S4. Structural accuracy and reliability metrics for prediction models. (A-D) Using the AlphaFold2_multimer software [73], models for actomyosin-7A were generated for four different actin isoforms, with five results produced for each isoform. Among these, we selected the model with the highest per-residue local distance difference test (pLDDT) score for each isoform, as elevated pLDDT values ensure that the models are highly reliable.

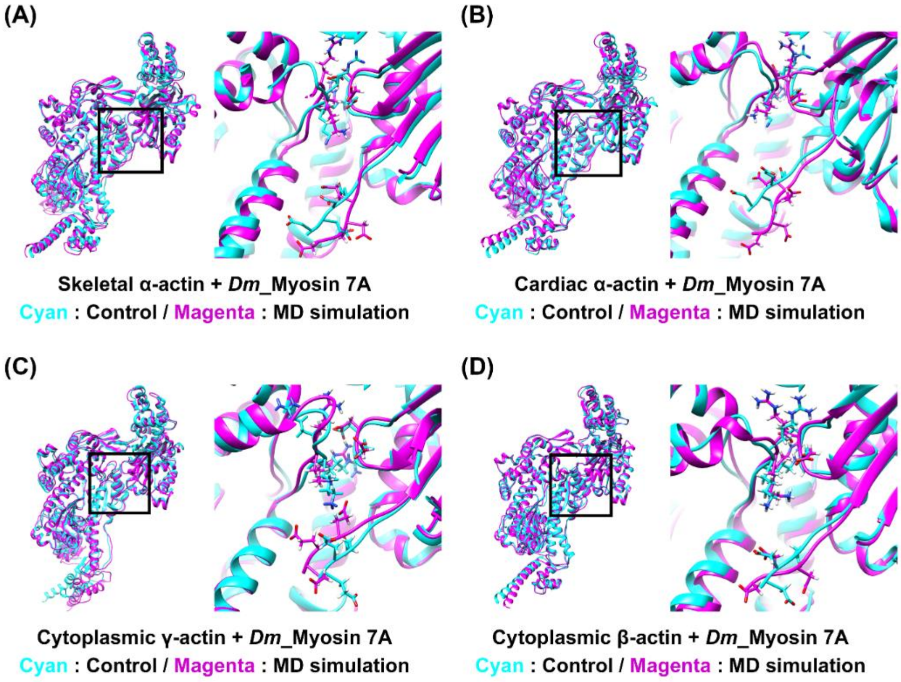

Fig. S5. Prediction of actin-loop 2 interaction using molecular dynamics (MD) simulation. (A-D) Due to the high flexibility of myosin loop 2, which interacts with the actin N-terminus, we aimed to observe changes in the interaction between loop 2 and the actin N-terminus through MD simulation. This MD simulation was run by GROMACS 2023.3 software [74]. However, four different actomyosin-7A complexes using the various actin isoforms did not reveal additional interactions with the actin N-terminus after the MD simulations, apart from the interaction with the actin subdomain 1 acidic patch D24, D25. Control actomyosin-7A model: cyan, Post-MD simulation actomyosin-7A model: magenta. Species codes: *Dm Drosophila melanogaster*.
